# Supplementary material for: Circular polarization-resolved ultraviolet photonic artificial synapse based on chiral perovskite
Source: Nat Commun. 2023 Nov 7;14:7179. doi: 10.1038/s41467-023-43034-3 (PMC10630371; doi:10.1038/s41467-023-43034-3)
Supplement: Supplementary file 1 — Supplementary Information [file 41467_2023_43034_MOESM1_ESM.pdf]

Supplementary Information

Circular polarization-resolved ultraviolet photonic artificial synapse based on chiral perovskite

Qi Liu[1†], Qi Wei[1†], Hui Ren[1], Luwei Zhou[1], Yifan Zhou[1], Pengzhi Wang[1], Chenghao Wang[1], Jun Yin[1], Mingjie Li[1,2,3]*

1. Department of Applied Physics, The Hong Kong Polytechnic University, Hung Hom, Kowloon, Hong Kong, China

2. Shenzhen Research Institute, The Hong Kong Polytechnic University Shenzhen, Guangdong, 518057, China

3. Photonics Research Institute, The Hong Kong Polytechnic University, Hung Hom, Kowloon, Hong Kong, China

[†] These authors contributed equally

*Email: ming-jie.li@polyu.edu.hk

Catalogue

Supplementary Fig. 1 | Molecular structures of 1D-S and 1D-R chiral perovskites.
Supplementary Fig. 2 | Crystal characteristic of H-PVKs.
Supplementary Fig. 3 | Characterization of H-PVK films.
Supplementary Fig. 4 | AFM characterization of H-PVK films.
Supplementary Fig. 5 | Absorbance spectra of H-PVK/SWNTs measured under LCP condition.
Supplementary Fig. 6 | Calculation of anisotropy factor of CD.
Supplementary Fig. 7 | Preparation for SWNTs fabrication.
Supplementary Fig. 8 | Raman characteristic of SWNTs.
Supplementary Fig. 9 | AFM characteristic of dip-coating SWNTs on SiO2/Si substrate.
Supplementary Fig. 10 | Schematic process for the preparation of two-terminal H-PVK/SWNTs heterobilayer devices.
Supplementary Fig. 11 | Surface morphology of SWNTs in top electrode channel.
Supplementary Fig. 12 | Band structure of H-PVK.
Supplementary Fig. 13 | Band alignments of H-PVK and SWNTs before and after connection. (Evac is the vacuum energy level, EF is the Fermi energy level).
Supplementary Fig. 14 | Chiroptical characterization of SWNTs.
Supplementary Fig. 15 | Wavelength-dependent characteristics of 1D-S/SWNTs heterostructure.
Supplementary Fig. 16 | The ambient stability of the heterostructures.
Supplementary Fig. 17 | DFT calculation models.
Supplementary Fig. 18 | Molecular structure of SWNTs/1D-R and related DFT calculation.
Supplementary Fig. 19 | Normalized TA dynamics.
Supplementary Fig. 20 | Photocarrier dynamics of 1DS and 1D-S/SWNTs.
Supplementary Fig. 21 | Photocarrier dynamics of 1D-R and 1D-R/SWNTs heterostructure.
Supplementary Fig. 22 | Near-IR TA measurements to probe the photoexcited hole transfer to SWNTs.
Supplementary Fig. 23 | Laser spike-numbers dependent change of band edge PB amplitudes probed at 375 nm and 5 ns of 1D-S (upper panel) and 1D-S/SWNT (lower panel) under 340 nm LCP/RCP excitation.
Supplementary Fig. 24 | Performance of H-PVK based device.
Supplementary Fig. 25 | Experimental optical setup for generating the CPL spikes.
Supplementary Fig. 26 | EPSC response under CPL with different handedness.
Supplementary Fig. 27 | Four times learning and forgetting process.
Supplementary Fig. 28 | Image memorization.
Supplementary Fig. 29 | Flow diagram of the implementation of the SNN.
Supplementary Table 1. Light-modulated photonic artificial synapse devices.

List of Acronyms

Acronyms	Definition
1D-S	(S-α-MBA)PbI_3
1D-R	(R-α-MBA)PbI_3
1D	One-Dimensional
2D	Two-Dimensional
3D	Three-Dimensional
AFM	Atomic Force Microscope
Au	gold
CPL	Circularly Polarized Light
CBM	Conduction Band Minimum
CD	Circular Dichroism
CNTs	Carbon Nanotubes
DFT	Density Functional Theory
EPSC	Excitatory Postsynaptic Current
FRRT	Fluorescence Resonance Energy Transfer
GSB	Ground-State Bleaching
H-PVK	Helical One-Dimensional Chiral Perovskite
LCP	Left-Handed Circularly Polarized Light
MNIST	Modified National Institute of Standards and Technology
PVK	Perovskite
PB	Photobleaching
PAS	Photonic Artificial Synapse
PDOS	Projected Density of States
RMS	Root Means Square
RCP	Right-Handed Circularly Polarized Light
SWNTs	Single-wall Carbon Nanotubes
Si	Silicon
SiO_2	Silicon dioxide
SNNs	Spiking Neural Networks
SEM	Scanning Electron Microscope
TA	Transient Absorption
Ti	Titanium
UPS	Ultraviolet photoelectron spectroscopy
VBM	Valence Band Maximum
XRD	X-ray Diffraction

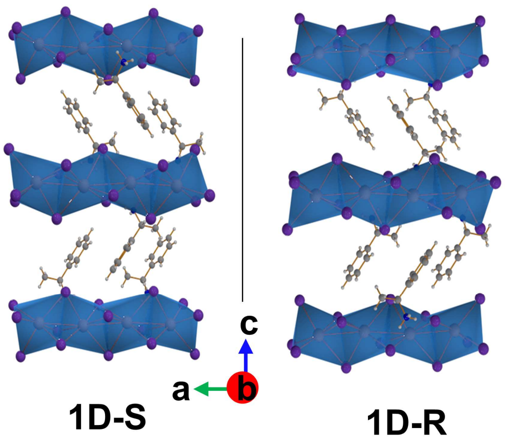

Supplementary Fig. 1 | Molecular structures of 1D-S and 1D-R chiral perovskites. Crystal structure of 1D-S and 1D-R from the view along *b* direction. In the molecular structure, the enantiomers (*R*- and *S*-*α*-MBA) remain in close proximity to the vertices of $(PbI_6)^{4-}$ octahedron, allowing for intense interactions between chiral organic and achiral inorganic parts.

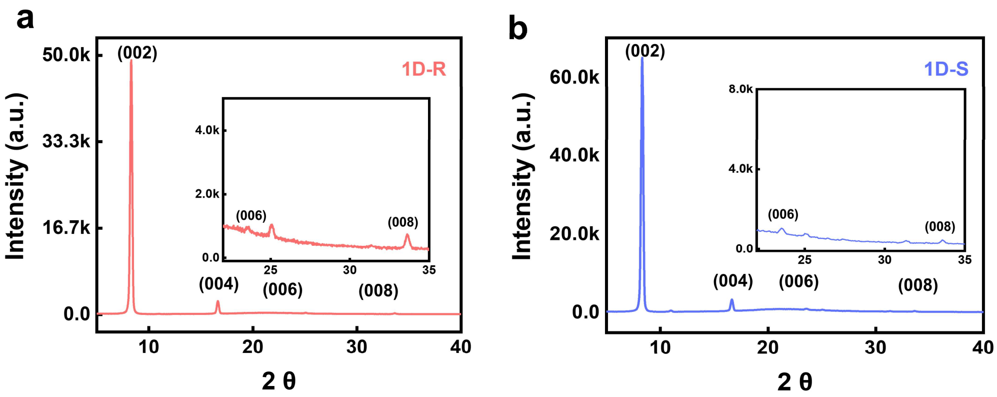

Supplementary Fig. 2 | Crystal characteristic of H-PVKs. (a), (b) XRD patterns of 1D-R and 1D-S films. The insets show the enlarged view of the XRD result range from 22° to 35°. XRD patterns of 1D-R and 1D-S films with the diffraction peaks of (002), (004), (006), and (008) suggest that the growth of spin-coated H-PVK features a preferred orientation along c-axis.

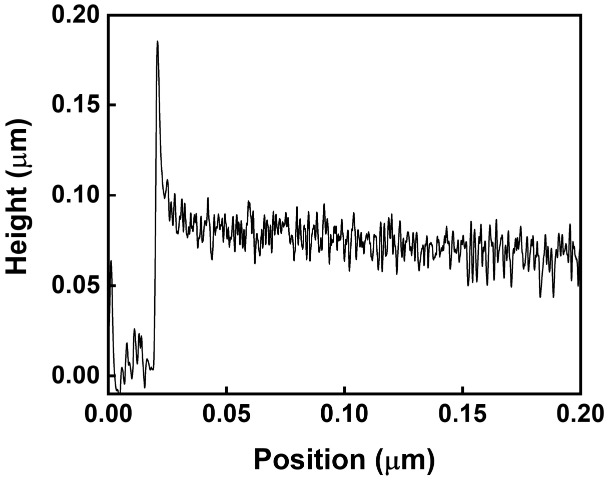

Supplementary Fig. 3 | Characterization of H-PVK films. Thickness of H-PVK film measured by surface profiler. The deposited H-PVK film has a thickness of approximately 70 nm.

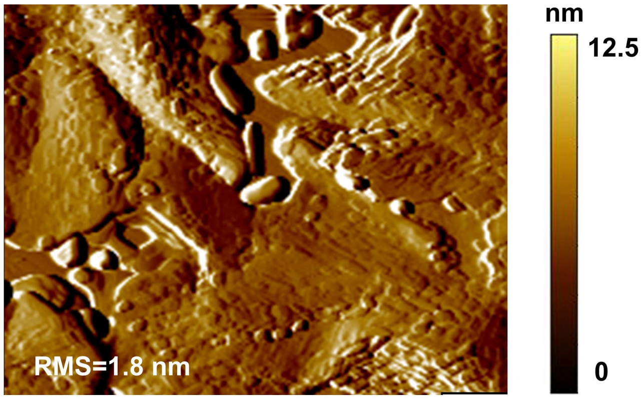

Supplementary Fig. 4 | AFM characterization of H-PVK films. AFM characteristic of spin-coating H-PVK film. The deposited H-PVK film shows a root means square (RMS) of 1.8 nm.

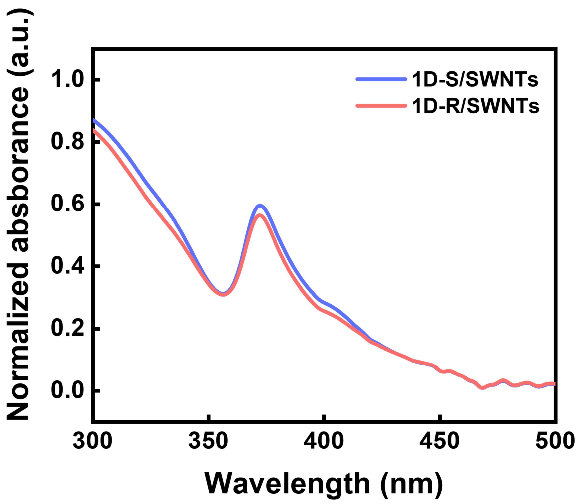

Supplementary Fig. 5 | Absorbance spectra of H-PVK/SWNTs measured under LCP condition.

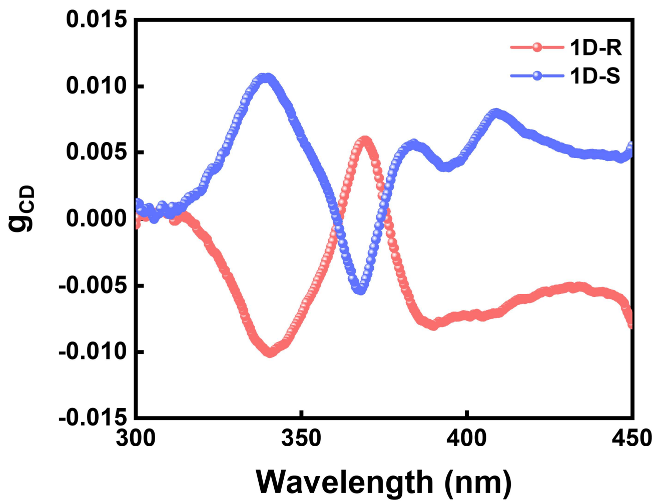

Supplementary Fig. 6 | Calculation of anisotropy factor of CD. The g_{CD} spectra of 1D-R and 1D-S films.

The dissymmetry of absorption can be calculated by the equation:

$$g_{CD} = \frac{\mathrm{CD[mdeg]}}{32980 \times \mathrm{absorbance}}$$

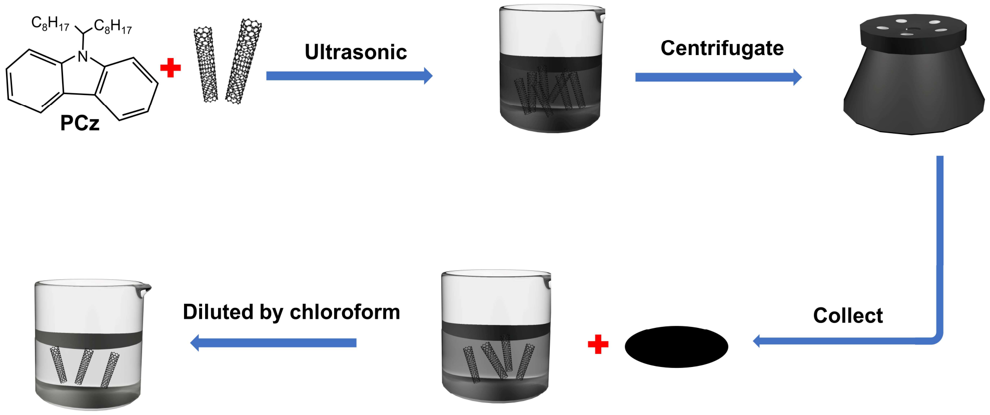

Supplementary Fig. 7 | Preparation for SWNTs fabrication. The schematic process for the preparation of semiconducting SWNT.

AP-SWNT has a typical diameter distribution with a peak value of 1.55 ± 0.1 nm and a bundle length of 1- 5 µm. High-purity semiconducting SWNTs are sorted by mixing dispersant PCz (5 mg) with AP-SWNT (5 mg) in 20 ml toluene, followed by ultrasonic for 1 h, and then centrifugation at 15,000 g for 1 h to remove CNT bundles and insoluble substances. Finally, the supernatants were collected and re-diluted in chloroform.

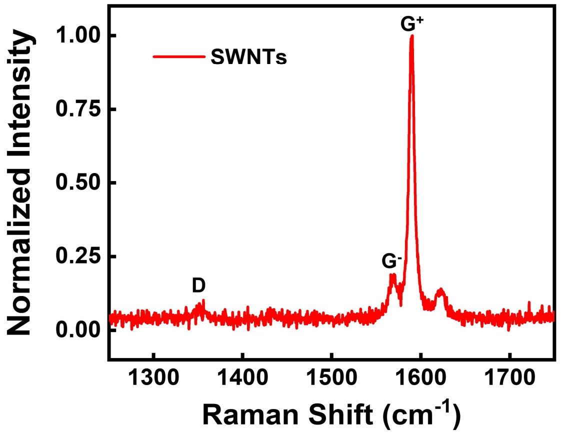

Supplementary Fig. 8 | Raman characteristic of SWNTs. Raman spectrum of SWNTs dip coating on SiO_2/Si substrate. Clear observation of G bands (G^+ and G^-) and D bands from Raman spectroscopy suggests the successful transfer of semiconducting SWNTs onto the Si substrate.

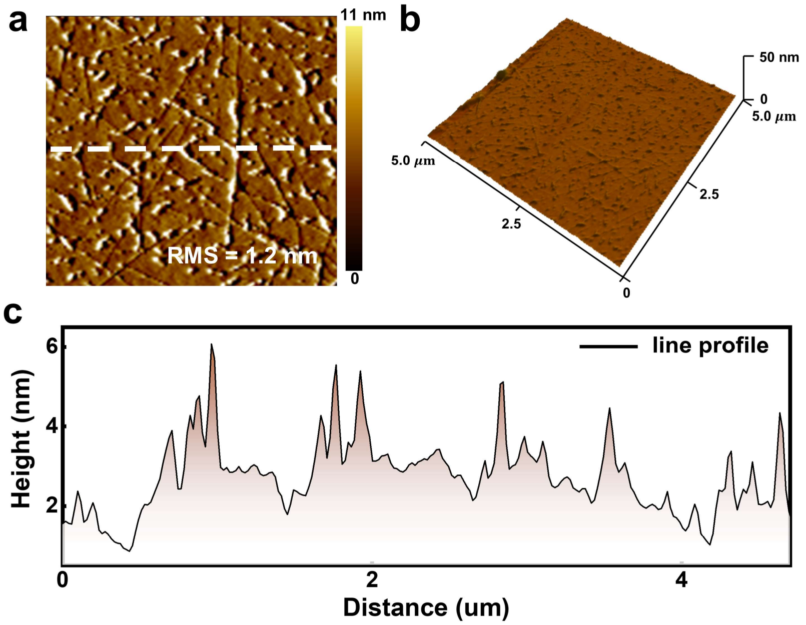

Supplementary Fig. 9 | AFM characteristic of dip-coating SWNTs on SiO_2/Si substrate. (a), (b) 2D and 3D AFM (5 × 5 μm) images of SWNTs network. **c** Extracted height data along white dash line in **a**. The calculated RMS from AFM measurements of the SWNTs surface is 1.2 nm, indicating a uniform SWNTs network is transferred onto the wafer with thickness of around 6 nm, indicating that a high-quality SWNTs network is obtained by the dip-coating method.

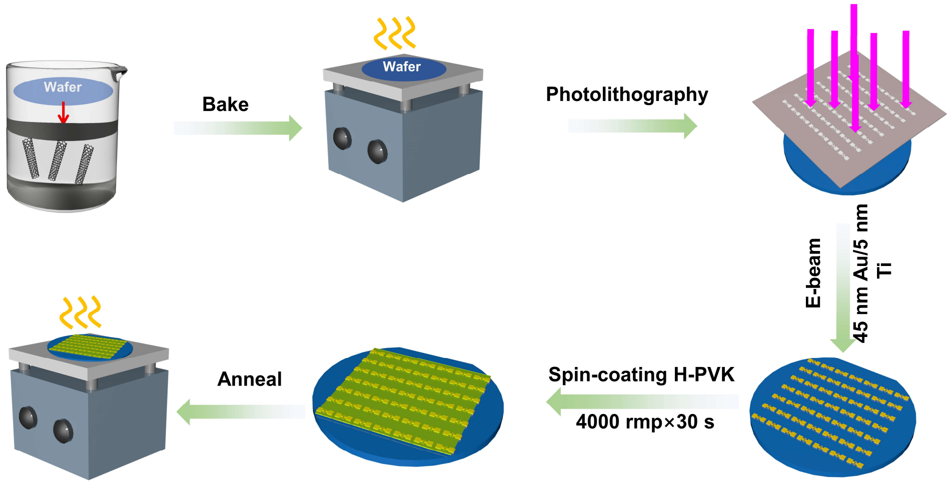

Supplementary Fig. 10 | Schematic process for the preparation of two-terminal H-PVK/SWNTs heterobilayer devices. The SWNTs film was formed by immersing the UV-ozone plasma-treated substrate (SiO_2/Si) into a semiconducting SWNTs solution for 24 hours, followed by a heating at 120 ℃ for 15 min. Then the electrodes (45 nm Au/5 nm Ti) were defined by typical photolithography, electron-beam evaporation, and a lift-off process. Next, the H-PVK film was spin-coated onto the patterned substrate with a spin rate of 4000 rpm for 30 s, followed by annealing at 95 ℃ for 15 min.

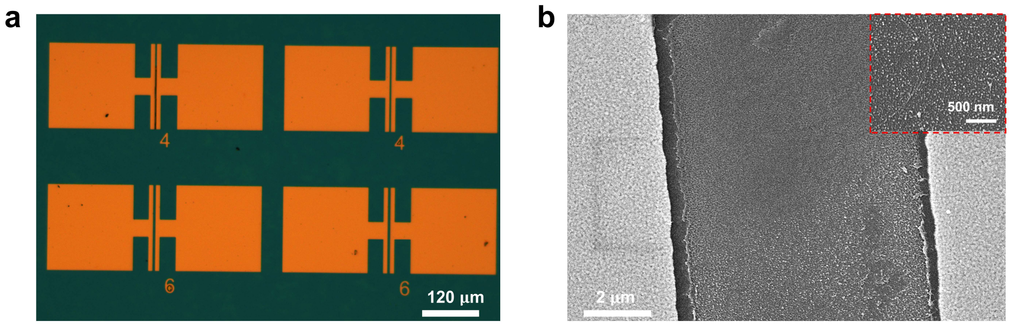

Supplementary Fig. 11 | Surface morphology of SWNTs in top electrode channel. (a) Optical image of partially selected Au electrodes array. (b) The SEM image of semiconducting SWNTs dip-coated in channel of top electrodes. The inset shows the enlarged part of the channel.

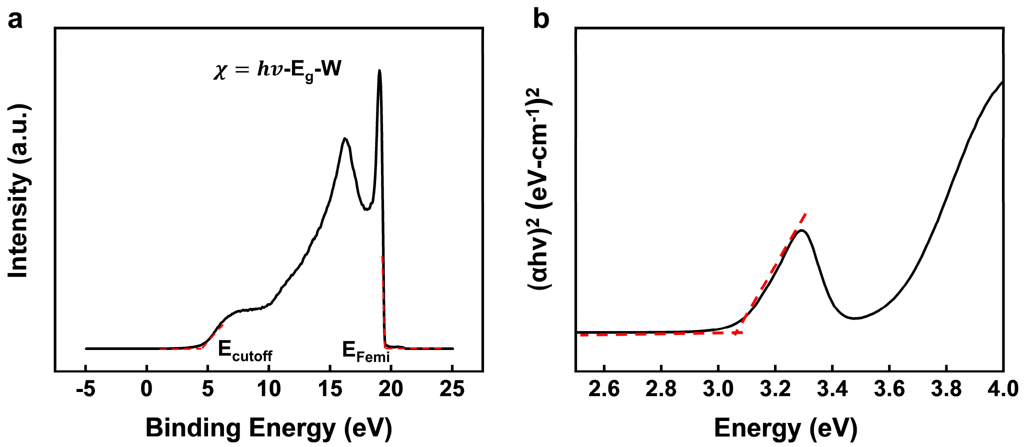

Supplementary Fig. 12 | Band structure of H-PVK. (a) UPS spectra of H-PVK. (b) The bandgap of H-PVK calculated by employing Tauc's plot of $(\alpha hv)^2$ versus energy.

The electron affinity is calculated by the equation:

$$\chi = hv\text{-}E_g\text{-}W$$

where hv is the energy of the He-I source (21.2 eV), W is the width of the UPS spectrum, and E_g is the bandgap of the H-PVK.

The direct bandgap of the H-PVK is calculated by the absorption-derivative method and the Tauc's equation as follow

$$B_1(Ahv)^2 + E_g = hv$$

where B_1 is the constant, A is the absorbance, hv is the photo energy, and E_g is the bandgap.

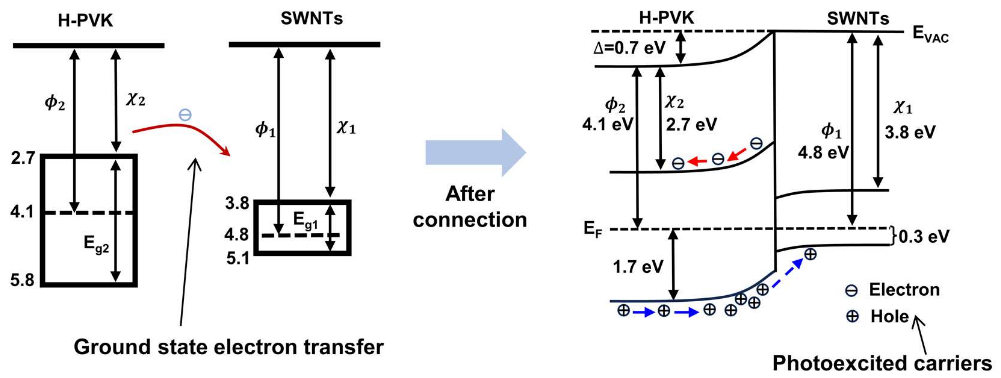

Supplementary Fig. 13 | Band alignments of H-PVK and SWNTs before and after connection. (E_{vac} is the vacuum energy level, E_F is the Fermi energy level).

Once H-PVK and SWNTs are connected, ground-state electron transfer occurs from PVK (with a low work function, higher E_F, from UPS) to SWNTs (with a high work function, lower E_F). This transfer continues until the Fermi levels align across the interface under equilibrium conditions, and results in interfacial energy band bending. Consequently, an electron depletion region can form at the H-PVK side, creating a potential barrier of 0.7 eV. This potential barrier is larger than the conduction band offset of 0.4 eV, making it unfavorable for photoexcited electron transfer from H-PVK to SWNTs.

Note that despite the fact that perovskite bandgap energy (including our chiral perovskites) is typically larger than that of SWNTs, no FRET energy transfer (requiring dipole-dipole interaction) or Dexter-like energy transfer (requiring wavefunction overlap) from perovskite film to SWNTs was reported. Since FRET or Dexter-like energy transfer requires the donor and accepter very close ~ 1-2 nm, which is usually occurs between quantum dot/SWNTs[1]. In our perovskite/SWNT heterostructure, the perovskite film is ~70 nm (Fig. S3), therefore, FRET or Dexter-like energy transfer from perovskite film to SWNTs should not be efficient.

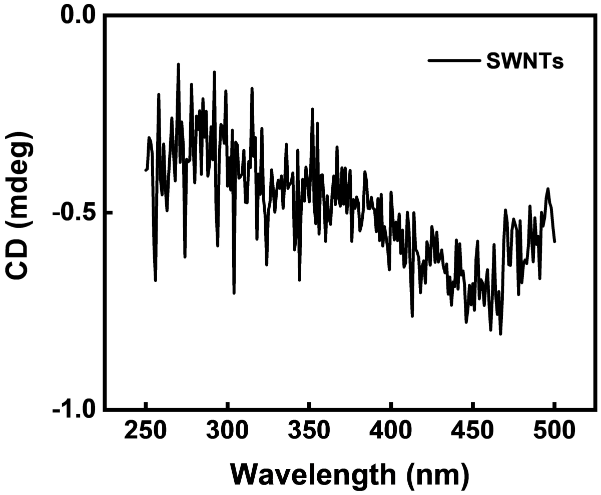

Supplementary Fig. 14 | Chiroptical characterization of SWNTs. CD spectra of SWNTs network on quartz substrate.

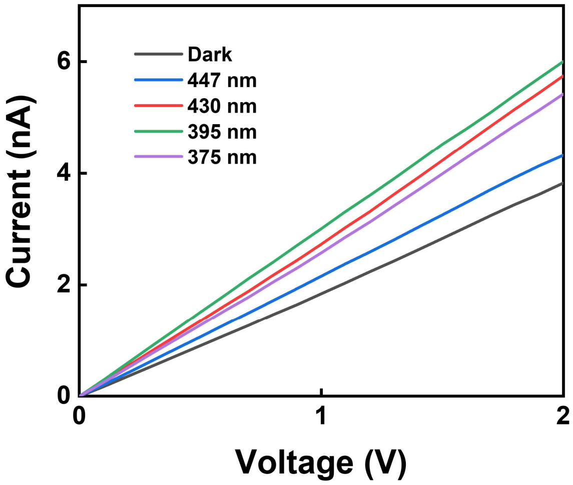

Supplementary Fig. 15 | Wavelength-dependent characteristics of 1D-S/SWNTs heterostructure. Photo-response behaviors of 1D-S/SWNTs under dark, and continuous wave LCP light with a wavelength of 375 nm, 395 nm, 430 nm, and 447 nm (10 μW cm^{-2}).

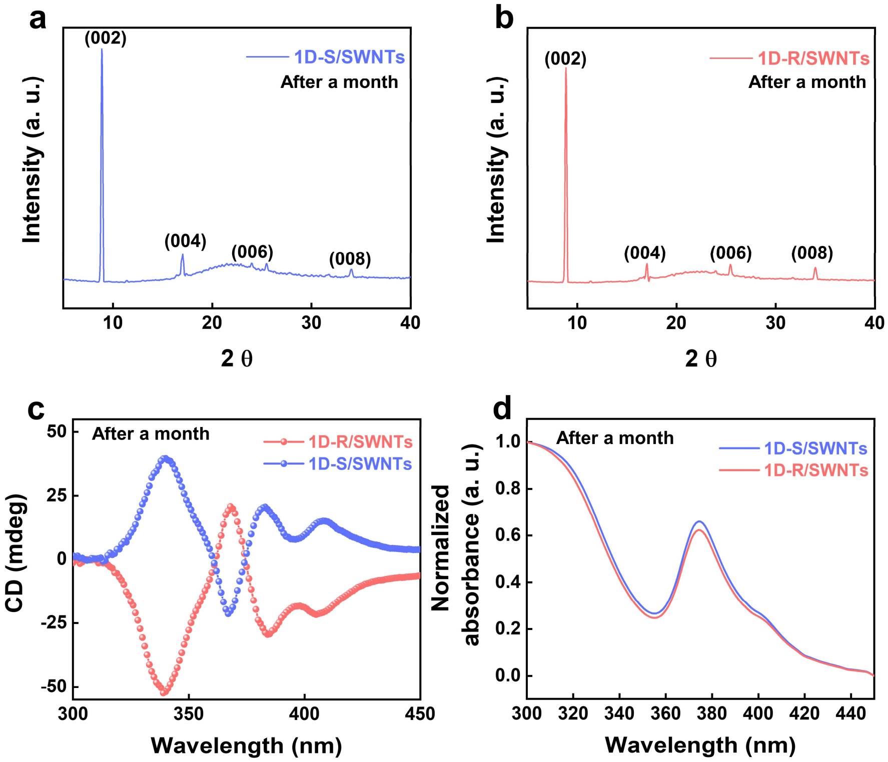

Supplementary Fig. 16 | The ambient stability of the heterostructures. XRD patterns for (a) 1D-R/SWNTs and (b) 1D-S/SWNTs. (c) CD spectra and (d) absorption spectra of heterostructure over a month in ambient.

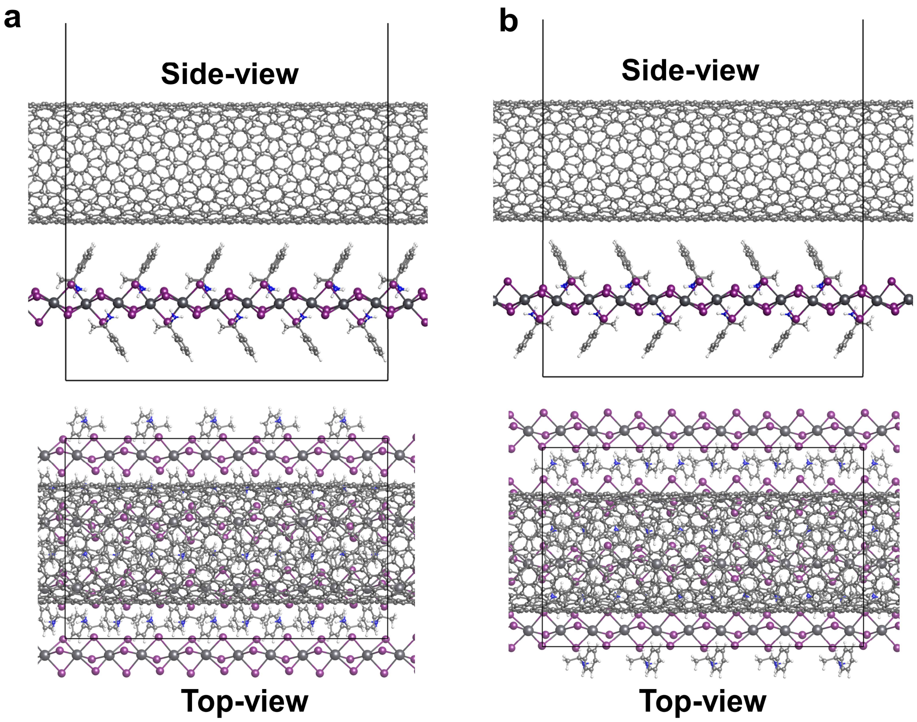

Supplementary Fig. 17 | DFT calculation models. Side-view and top-view of optimized interfacial structures for (a) 1D-R/SWNTs and (b) 1D-S/SWNTs.

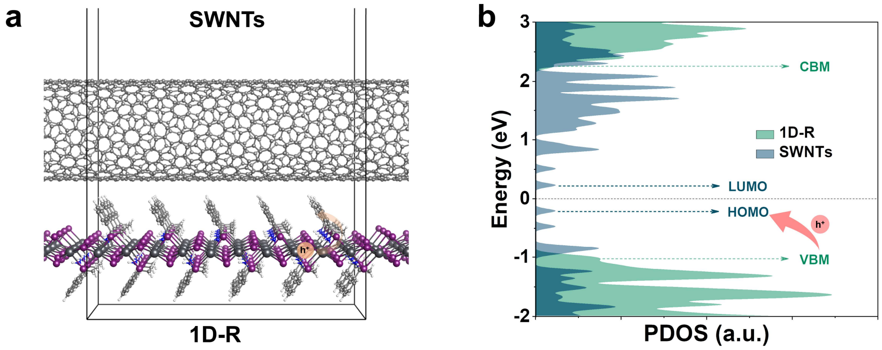

Supplementary Fig. 18 | Molecular structure of SWNTs/1D-R and related DFT calculation. (a) Optimized interfacial structures. (b) PDOS of 1D-R/SWNTs heterostructure.

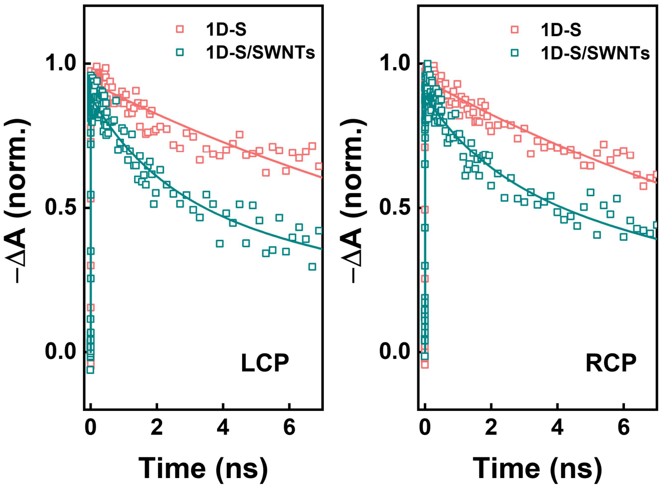

Supplementary Fig. 19 | Normalized TA dynamics. GSB dynamics of 1D-S and 1D-S/SWNTs under 340 nm LCP (left) and RCP (right) pump beams.

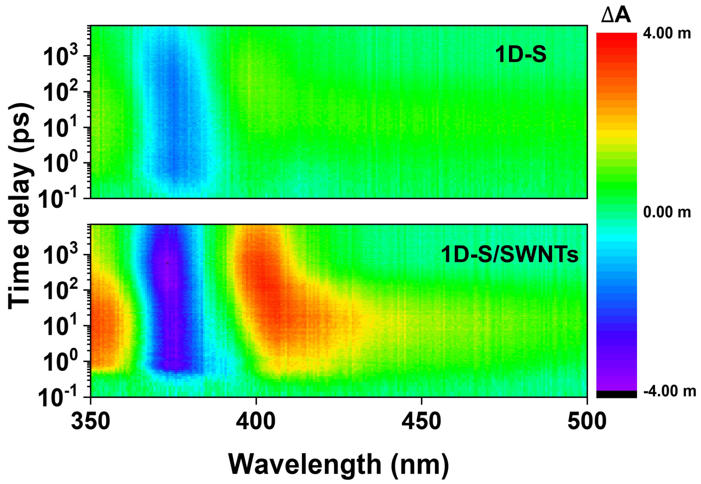

Supplementary Fig. 20 | Photocarrier dynamics of 1D-S and 1D-S/SWNTs. Time-resolved TA mapping of 1D-S and 1D-S/SWNTs measured under 340 nm RCP pump beam.

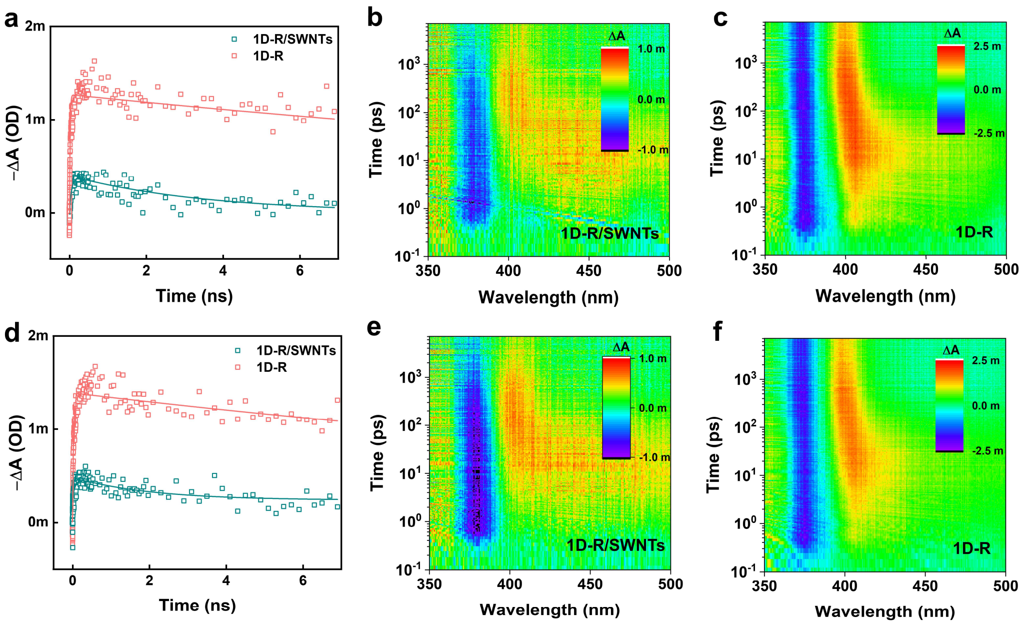

Supplementary Fig. 21 | Photocarrier dynamics of 1D-R and 1D-R/SWNTs heterostructure. (a) Band edge ground-state bleaching (GSB) dynamics of 1D-R and 1D-R/SWNTs under 340 nm LCP excitation pump beam ($\eta_i = 54\%$). Time-resolved TA mapping of (b) H-PVK and (c) 1D-R/SWNTs under LCP pump beam. (d) GSB dynamics of 1D-R and 1D-R/SWNTs under 340 nm RCP excitation pump beam ($\eta_i = 67\%$). Time-resolved TA mapping of (e) 1D-R and (f) 1D-R/SWNTs measured under RCP pump beam.

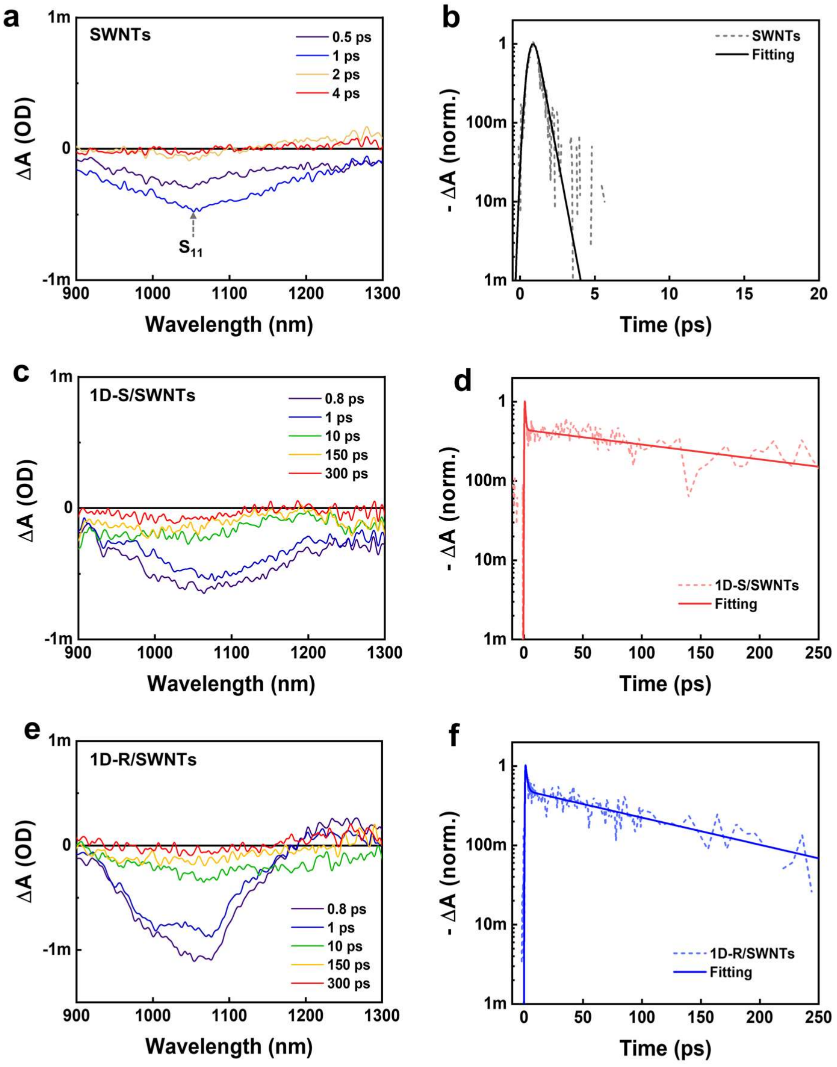

Supplementary Fig. 22 | Near-IR TA measurements to probe the photoexcited hole transfer to SWNTs. (a, c, e) TA spectra at various delay time after subtraction of the background signal and (b, d, f) normalized GSB dynamics probed at ~1050 nm for SWNTs, 1D-R/SWNTs, and1D-R/SWNTs, respectively. The excitation wavelength is 340 nm.

The photobleaching (PB) band with peak at ~1050 nm arising from the stating filling of S_{11} exciton transition along with a fast decay lifetime (< 1ps) is observed in our SWNTs sample (Supplementary Figs. 22a-b), which is consistent with previous results[1-3]. After addition of our chiral perovskites, an additional long-lived decay component probed at 1050 nm is observed in heterostructures (Supplementary Figs. 22c-f), which arises from the stating filling of the separated holes. These observations are consistent with previous reports of the long-lived separated holes in SWNTs[1, 4].

Note that the fast decay from S_{11} exciton is still existing in TA dynamics of heterostructures, which could be due to the energy transfer and/or the direct excitation of SWNTs by the pump laser. However, even if there are some short-lived excitons in SWNTs, they cannot contribute

to the photocurrent due to the fast recombination, it thus could only affect the quantum efficiency of device, and won't affect the fundamental concept and function of our LCP/RCP resolvable artificial synapse devices.

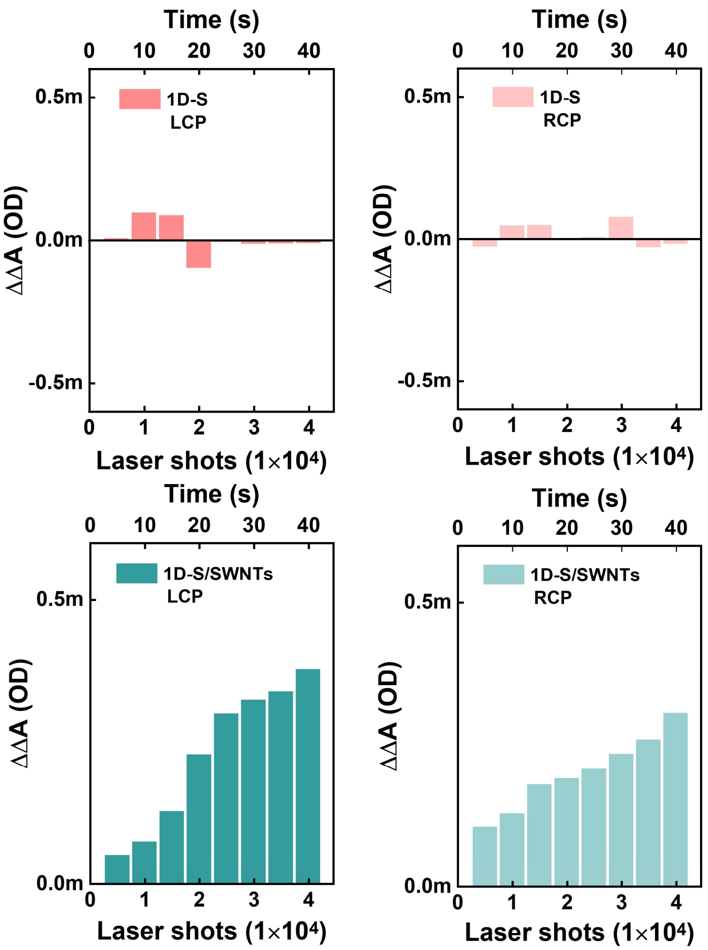

Supplementary Fig. 23 | Laser spike-numbers dependent change of band edge PB amplitudes probed at 375 nm and 5 ns of 1D-S (upper panel) and 1D-S/SWNTs (lower panel) under 340 nm LCP/RCP excitation.

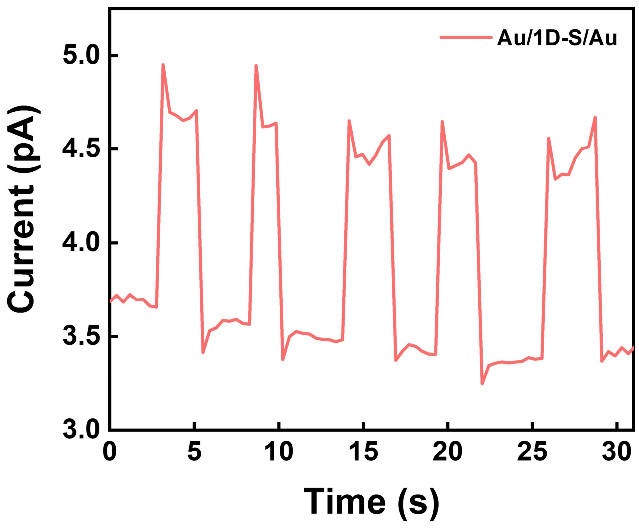

Supplementary Fig. 24 | Performance of H-PVK based device. Time-current response of planer Au/1D-S/Au under 395 nm LCP beam with an intensity of 10 μW cm^{-2}.

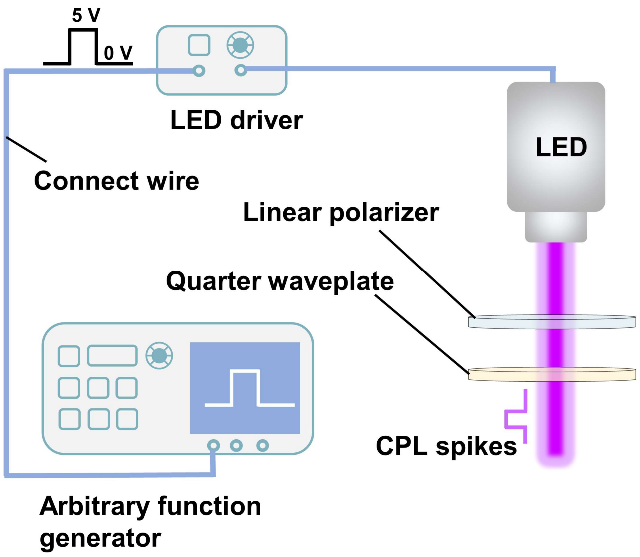

Supplementary Fig. 25 | Experimental optical setup for generating the CPL spikes. An arbitrary function generator, utilized for pulse width modulation, is connected to the LED driver responsible for controlling the LED. When the input voltage reaches the threshold of 5 V, the LED driver switches the output current to the level predetermined by the knob on the front of the unit, indicating that the LED is in the ON state. Conversely, when the input voltage falls below the threshold, the LED remains in the OFF state. After passing through the linear polarizer and quarter waveplate, the light spikes result in the generation of CPL spikes.

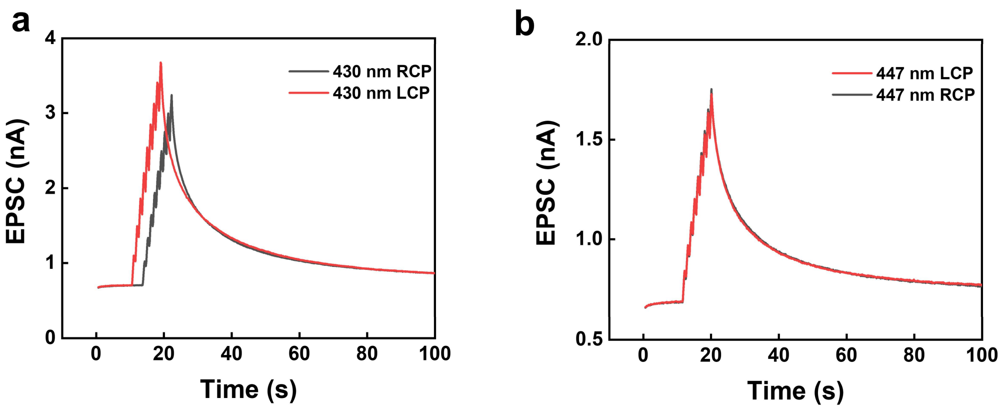

Supplementary Fig. 26 | EPSC response under CPL with different handedness. EPSCs in response to multiple RCP/LCP pulses with wavelengths of (a) 430 nm and (b) 447 nm, respectively.

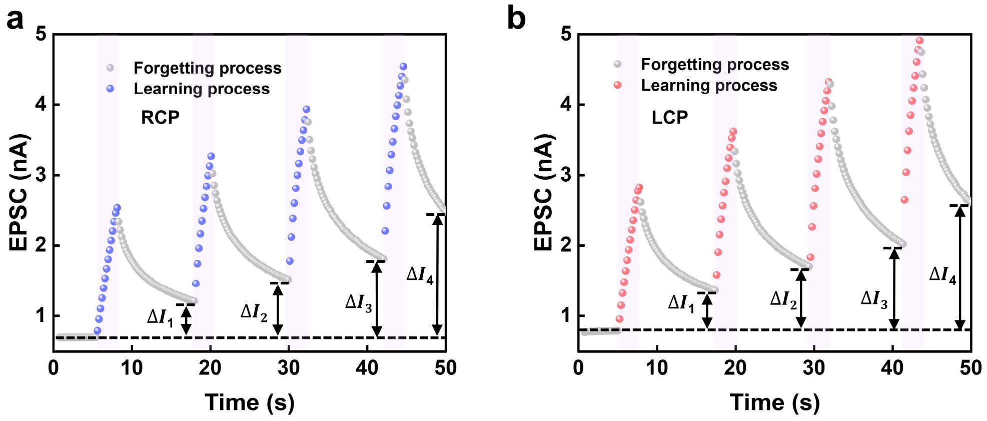

Supplementary Fig. 27 | Four times learning and forgetting process. Learning and forgetting behaviors simulated under 395 nm (a) RCP and (b) LCP illumination with an interval of 2 s and intensity of 10 μW cm^{-2} for 1D-S/SWNTs based PAS.

Learning and forgetting behaviors are simulated by generating four sets of LCP and RCP spikes. The EPSC can be potentiated by the CPL spikes and then decay over time, reaching an intermediate current level once the excitation is terminated. Notably, the 1D-S based PAS shows relatively higher EPSC response and slower decay under LCP illumination, implying that LCP is a more effective modulation method. The overall synaptic behavior aligns with the phenomenon that learned information tends to be forgotten over time. By implementing the relearning process, the EPSC values (ΔI_n, where n is the learning times) rapidly increases, suggesting the memory can be strengthened by repeating the learning process.

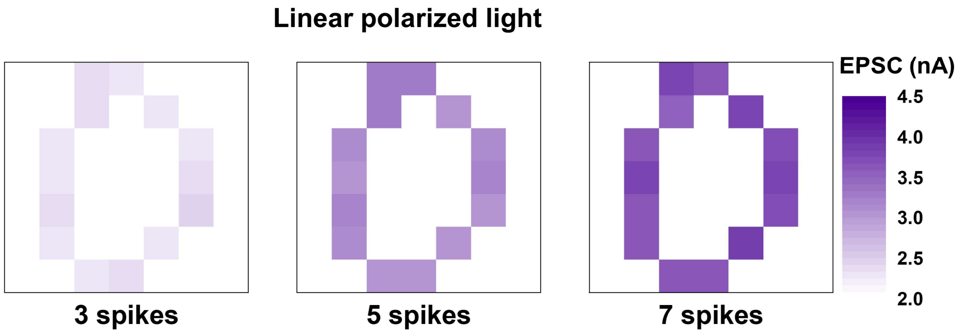

Supplementary Fig. 28 | Image memorization. Measured weights of the object images after a sequence of 395 nm linear polarized light spikes (10 μW cm^{-2}, 1 Hz).

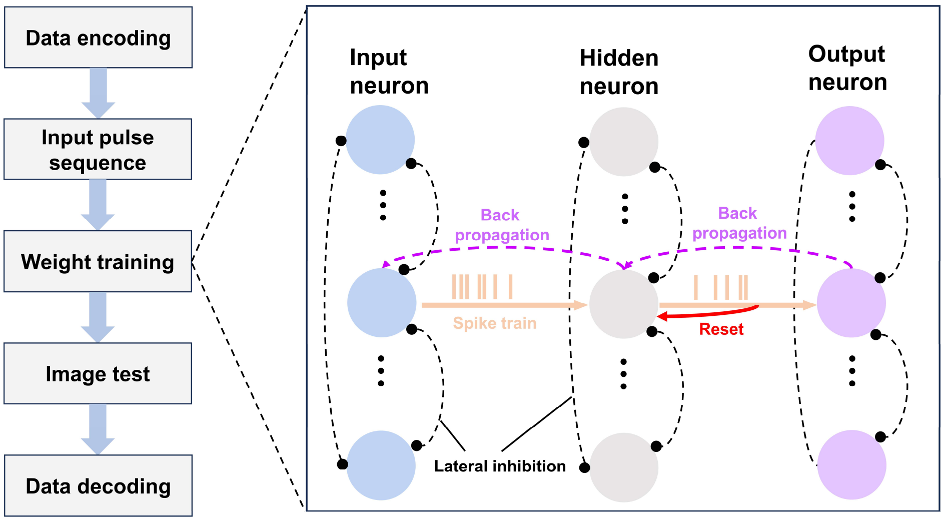

Supplementary Fig. 29 | Flow diagram of the implementation of the SNNs.

Each dataset is encoded as a spike train that conforms to the Poisson equation, and then input to the neurons. The fire rate of neurons relies on the intensity of the corresponding pixel in the dataset image. For an MNIST image, if the spiking probability of a white pixel is 100%, and a black pixel never generates a spike. In the training process, the membrane potential of each output neuron accumulates by the input spikes and weight from other connecting synapses. When the membrane potential value of one output neuron exceeds the threshold, the neuron fires and releases a spike to its next connections, and its membrane potential are reset. The fired neuron will also prevent other neurons from firing by lateral inhabitation. Within a period, the neuron keeps refractory state and cannot be fired. Moreover, the corresponding synapses that contributes to the firing result will be strengthened while synapses without contribution for the fire will be weakened. After the training, every category dataset is labeled with fixed threshold and training weights. In the test process, the images are input to the trained network, the output neurons are labeled to the pattern categories in terms of their most firing times to the corresponding input dataset.

Supplementary Table 1. Light-modulated photonic artificial synapse devices

Light polarization	Range/ Wavelength	Materials	Working Mechanism	Maximum Photoresponsivity (V_g = 0 V)	Object imaging and memorization	Ref.
Unpolarized	UV (345 nm)	Biopolymer	Charge trapping	-	No	5
Unpolarized	UV (365 nm)	$CsPbBr_3$ nanocrystals	Resistive switching	-	No	6
Unpolarized	UV (375 nm)	VO_2	Phase transition	-	Yes	7
Unpolarized	Visible (660 nm)	MoS_2	Charge trapping	< 100 mA/W	Yes	8
Unpolarized	UV (365 nm)	Perylene/ Graphene oxide	Charge trapping	30 mA/W	Yes	9
CPL	Visible (635 nm)	Chiral Au nanoparticles/IGZO	Charge trapping	0.98 mA/W (LCP) 1.08 mA/W (RCP)	No	10
**CPL**	**UV (395 nm)**	**Chiral perovskite (1D-S/SWNTs)**	**Charge transfer**	**240 mA/W (LCP)**	**Yes**	**This work**
		**Chiral perovskite (1D-R/SWNTs)**		**237 mA/W (RCP)**		

Supplementary Reference

1. Kang, H. S. *et al*. Long-lived charge separation at heterojunctions between semiconducting single-walled carbon nanotubes and perylene diimide electron acceptors. *J. Phys. Chem. C* **122**, 14150-14161 (2018).
2. Huang, L., Pedrosa, H. N., Krauss, T. D. Ultrafast ground-state recovery of single-walled carbon nanotubes. *Phys. Rev. Lett.* 93, 017403 (2004).
3. Graham, M. W. *et al.* Exciton dynamics in semiconducting carbon nanotubes. *J. Phys. Chem. B* **115**, 5201-5211 (2011).
4. Ellingson, R. J. *et al.* Ultrafast photoresponse of metallic and semiconducting single-wall carbon nanotubes. *Phys. Rev. B* **71**, 115444 (2005).
5. Lv, Z. *et al.* Mimicking neuroplasticity in a hybrid biopolymer transistor by dual modes modulation. *Adv. Funct. Mater*. **29**, 1902374 (2019).
6. Wang, Y. *et al.* Memristor-based biomimetic compound eye for real-time collision detection. *Nat. Commun.* **12**, 5979 (2021).
7. Li, G. *et al.* Photo-induced non-volatile VO_2 phase transition for neuromorphic ultraviolet sensors. *Nat. Commun.* **13**, 1729 (2022).
8. Liao, F. *et al.* Bioinspired in-sensor visual adaptation for accurate perception. *Nat. Electron.* **5**, 84-91 (2022).
9. Zhang, H.-S. *et al.* Co-assembled perylene/graphene oxide photosensitive heterobilayer for efficient neuromorphics. *Nat. Commun*. **13**, 4996 (2022).
10. Namgung, S. D. *et al.* Circularly polarized light-sensitive, hot electron transistor with chiral plasmonic nanoparticles. *Nat. Commun.* **13**, 5081 (2022).
